# Predicting delayed anxiety and depression in patients with gastrointestinal cancer

**DOI:** 10.1038/sj.bjc.6690082

**Published:** 1999-02

**Authors:** K Nordin, B Glimelius

**Affiliations:** Department of Oncology, University Hospital, Uppsala, S-751 85, Sweden; Centre for Caring Sciences, Uppsala University, Uppsala Science Park, Uppsala, S-751 83, Sweden

**Keywords:** cancer, depression, anxiety, prediction

## Abstract

The aim of this study was to examine the possibility of predicting anxiety and depression 6 months after a cancer diagnosis on the basis of measures of anxiety, depression, coping and subjective distress associated with the diagnosis and to explore the possibility of identifying individual patients with high levels of delayed anxiety and depression associated with the diagnosis. A consecutive series of 159 patients with gastrointestinal cancer were interviewed in connection with the diagnosis, 3 months (non-cured patients only) and 6 months later. The interviews utilized structured questionnaires assessing anxiety and depression [Hospital Anxiety and Depression (HAD) scale], coping [Mental Adjustment to Cancer (MAC) scale] and subjective distress [Impact of Event (IES) scale]. Patient anxiety and depression close to the diagnosis were found to explain approximately 35% of the variance in anxiety and depression that was found 6 months later. The addition of coping and subjective distress measures did little to improve that prediction. A model using (standardized) cut-off scores of moderate to high anxiety, depression (HAD) and intrusive thoughts (IES subscale) close to the diagnosis to identify patients at risk for delayed anxiety and depression achieved a sensitivity of 75% and a specificity of 98%. Levels of anxiety and depression at diagnosis predicted a similar status 6 months later. The results also indicated that the HAD scale in combination with the IES intrusion subscale may be used as a tool for detecting patients at risk of delayed anxiety and depression. © 1999 Cancer Research Campaign


British Journal of Cancer (1999) 79(3/4), 525–529
© 1999 Cancer Research Campaign
Article no. bjoc.1998.0082

### Predicting delayed anxiety and depression in patients with gastrointestinal cancer

**K Nordin[1,2] and B Glimelius[1]**

[1]Department of Oncology, University Hospital, S-751 85 Uppsala, Sweden; [2]Centre for Caring Sciences, Uppsala University, Uppsala Science Park, S-751 83 Uppsala, Sweden

**Summary** The aim of this study was to examine the possibility of predicting anxiety and depression 6 months after a cancer diagnosis on the basis of measures of anxiety, depression, coping and subjective distress associated with the diagnosis and to explore the possibility of identifying individual patients with high levels of delayed anxiety and depression associated with the diagnosis. A consecutive series of 159 patients with gastrointestinal cancer were interviewed in connection with the diagnosis, 3 months (non-cured patients only) and 6 months later. The interviews utilized structured questionnaires assessing anxiety and depression [Hospital Anxiety and Depression (HAD) scale], coping [Mental Adjustment to Cancer (MAC) scale] and subjective distress [Impact of Event (IES) scale]. Patient anxiety and depression close to the diagnosis were found to explain approximately 35% of the variance in anxiety and depression that was found 6 months later. The addition of coping and subjective distress measures did little to improve that prediction. A model using (standardized) cut-off scores of moderate to high anxiety, depression (HAD) and intrusive thoughts (IES subscale) close to the diagnosis to identify patients at risk for delayed anxiety and depression achieved a sensitivity of 75% and a specificity of 98%. Levels of anxiety and depression at diagnosis predicted a similar status 6 months later. The results also indicated that the HAD scale in combination with the IES intrusion subscale may be used as a tool for detecting patients at risk of delayed anxiety and depression.

**Keywords:** cancer; depression; anxiety; prediction

A high level of psychological distress is a normal reaction to a stressful event, e.g. a cancer diagnosis, most commonly soon after the event (Horowitz, 1993). The most common reactions in cancer patients are anxiety and depression (Derogatis et al, 1983; Glanz and Lerman, 1992). The prevalence figures for anxiety and depression disorders range from 0 to 49% (Spijker et al, 1997, for a review). Anxiety decreases over time, but there is usually no significant decrease in depression. Meta-analyses have shown that the average levels of anxiety and overall psychological distress in cancer patients during follow-up do not differ significantly from those of the normal population. However, the levels of depression are higher (Spijker et al, 1997).

In a preceding study (Nordin and Glimelius, 1997), we found low average levels of anxiety and depression in a group of newly diagnosed gastrointestinal cancer patients. However, despite low average levels, some individuals had high levels of either anxiety or depression, or both, not only soon after the diagnosis, but also during follow-up. For those in whom the distress does not decrease over time, psychological interventions may be necessary, and it is important to identify these patients as early as possible. The demonstrated lack of training and skills, in non-specialized clinicians and nurses, in detecting psychological distress in cancer patients (Maguire, 1992) suggests the need for a complementary method to identify patients who are at risk of psychological distress, and especially those who are at risk of prolonged distress.

Received 18 February 1998
Revised 18 May 1998
Accepted 4 June 1998

Correspondence to: K Nordin, Centre for Caring Sciences, Uppsala University, Uppsala Science Park, S-751 83 Uppsala, Sweden

In some studies, prediction of outcome by means of baseline anxiety and depression measured by the HAD scale has been attempted, but none of the studies concerned gastrointestinal cancer patients (Burton et al, 1995; Leigh et al, 1995). Several studies have also shown that cancer patients may benefit from psychological interventions (Watson, 1983; Andersen, 1992, Trijsburg et al, 1992). The use of cognitive–behavioural techniques is well established for the treatment of both anxiety and depression in cancer patients (Greer et al, 1992; Fawzy et al, 1993; Watson et al, 1996). In the study by Greer et al (1992), patients who received adjuvant psychological therapy reported significant improvements in various measures of psychological distress, especially anxiety. Watson et al (1996), however, found no significant changes in psychological distress before vs after a cognitive–behavioural treatment programme developed for cancer patients. One important difference between these two studies is that the patients selected for the Greer et al (1992) study suffered from high levels of psychological distress, whereas the patients in the study by Watson et al (1996) were all self-referred. In studies of psychological interventions with cancer patients recruited consecutively without any selection, it may be difficult to show any effects of the interventions. Andersen (1992) makes a distinction between psychological interventions for low, moderate and high morbidity risk patients. This distinction is important for the evaluation of interventions, since the rate of spontaneous recovery may vary with the risk of psychosocial morbidity (Andersen, 1992).

The aim of the present study was to investigate to what extent it is possible to predict elevated levels of anxiety and depression 6 months after the diagnosis in a group of gastrointestinal cancer patients. It may not be realistic to provide psychological support to all patients who want it, or even to all patients with moderate or high levels of anxiety and depression at diagnosis. However, it is

**Table 1** Number of patients (potentially cured/non-cured) interviewed at diagnosis and after 6 months

| | Diagnosis | 6 months |
|---|---|---|
| *n* | 82/77 | 69/31 |
| Colon | 41/8 | 37/4 |
| Rectum | 29/10 | 25/6 |
| Gastric | 11/21 | 6/14 |
| Biliary, pancreatic | 0/38 | 0/17 |
| Male | 48/33 | 41/14 |
| Female | 34/44 | 28/17 |
| Age (mean) | 68 (range 23–89) | 66 (range 23–89) |

**Table 2** Mean scores of HAD anxiety and depression at diagnosis and after 6 months

| | Diagnosis | | 6 months | | |
|---|---|---|---|---|---|
| | Mean | s.d. | Mean | s.d. | |
| All | | | | | |
| ($n$ = 151) | | | | | |
| Anxiety | 4.0 | 4.2 | | | |
| Depression | 4.4 | 3.9 | | | |
| ($n$ = 98) | | | | | |
| Anxiety | 3.6 | 3.6 | 3.0 | 2.8 | $P < 0.05$ |
| Depression | 3.7 | 3.3 | 3.0 | 2.7 | $P < 0.05$ |

**Table 3** Prediction of HAD anxiety and/or depression 6 months after diagnosis on the basis of anxiety, depression, subjective distress and coping close to the diagnosis

| | $R^2$ | β | s.e. (β) | *t* |
|---|---|---|---|---|
| HAD anxiety at 6 months | | | | |
| Anxiety | 0.31 | 0.27 | 0.10 | 2.7** |
| Depression | 0.35 | 0.25 | 0.10 | 2.5* |
| HAD depression at 6 months | | | | |
| Depression | 0.31 | 0.33 | 0.09 | 3.67** |
| Hopeless/helplessness | 0.34 | 0.23 | 0.10 | 2.3* |
| Sum of HAD anxiety and depression at 6 months | | | | |
| Depression | 0.37 | 0.67 | 0.15 | 4.47** |
| Intrusion | 0.41 | 0.18 | 0.08 | 2.25* |

*$P < 0.05$, **$P < 0.01$

important to identify those patients who continue to experience elevated levels of anxiety and depression several months after the diagnosis. The following specific research questions were raised.

(1) To what extent can anxiety and depression 6 months after diagnosis be predicted on the basis of measures of anxiety, depression, subjective distress and coping close to the diagnosis?
(2) Is it possible to identify individual patients with moderate or high levels of depression and anxiety 3 months (non-cured patients) or 6 months (potentially cured patients) after the diagnosis on the basis of the same factors?

### PATIENTS AND METHODS

### Patients

A consecutive series of newly diagnosed patients from the three hospitals in the county of Uppsala with gastrointestinal cancers (colon, rectum, gastric, pancreatic and biliary) were asked to participate. Patients were excluded who did not speak Swedish, had a very poor performance (Karnofsky performance status ≤30) or were senile or confused.

Data on age, gender, diagnosis and whether or not the patient was potentially cured (surgically inextirpable primary and/or metastatic non-resectable disease), obtained close to the diagnosis, were compiled from the medical records (Table 1).

### Study design

Patients who agreed to participate were interviewed as soon as their physical status permitted, or within 12 weeks from the date of the diagnostic biopsy or primary surgery (median 3 weeks). The patients who were considered not to be cured at diagnosis were interviewed again after 3 months because of their expected short survival. Both groups were interviewed after 6 months.

### Participation and compliance

Of 196 eligible patients, 159 (81%) were interviewed in connection with the diagnosis. Of those who did not participate, 30 chose not to do so, three were hard of hearing and four were not aware of their diagnosis. The non-participants (22 patients with colorectal cancer, ten with gastric and five with pancreatic/biliary cancer) were older (mean 78 years, range 51–88) than the participants (mean 67 years, range 23–89), and there were more women ($n$ = 24) than men ($n$ = 13) in this group. The number of patients interviewed on each different occasion is shown in Table 1. Of the non-cured patients not interviewed at 3 months, 27 had died, five were in too poor a condition, one did not want to participate and two could not be traced. At 6 months, a further 11 patients had died, one was in too poor a condition and one did not want to participate. Of the potentially cured patients not interviewed at 6 months, one had died, four were in too poor a condition and four did not want to participate.

### Measures

The interviews were based on structured questionnaires assessing coping, subjective distress, anxiety and depression.

#### *Coping*

The Mental Adjustment to Cancer (MAC) scale consists of 40 items constituting five subscales (coping styles): fighting spirit (16 items), hopeless/helplessness (six items), anxious preoccupation (nine items), fatalistic (eight items) and avoidance (one item) (Watson et al, 1988). Except for avoidance, these subscales show good psychometric properties (Watson et al, 1988; Greer et al, 1989). As suggested by Schwartz et al (1992), the avoidance subscale was eliminated from the analysis.

#### *Subjective distress*

The Impact of Event Scale (IES) assesses current subjective distress due to a specific life event (Horowitz et al, 1979; Horowitz, 1993), which in the present study is the distress due to the diagnosis of a cancer disease. It is a 15-item scale that measures intrusion (range 0–35), characterized by unbidden thoughts and/or images of

 © Cancer Research Campaign 1999

**Table 4** Number of patients who scored as 'doubtful cases' or 'cases' for HAD anxiety and depression after 3 or 6 months related to how they scored at diagnosis using the HAD scale alone (Model 1) or the HAD scale supplemented by IES intrusion (Model 2)

| Model | Scoring at diagnosis | | HAD 'doubtful cases' or 'cases' after 3 or 6 months[a] | |
|---|---|---|---|---|
| | | | Yes | No |
| 1. HAD 'doubtful cases' or 'cases' | Yes | 24 | 12 | 12 |
| | No | 89 | 2 | 87 |
| | | | Yes | No |
| 2. HAD 'doubtful cases' or 'cases' and above cut-off for IES intrusion | Yes | 16 | 12 | 4 |
| | No | 97 | 2 | 95 |

[a]Data for non-cured patients scored at 3 months and for potentially cured patients at 6 months.

the event, and avoidance (range 0–40), characterized by denial of meanings and consequences of the event. The level of distress is considered to be low at a score of 0–8, medium at 9–19 and high at 20 or above (Horowitz, 1993). The patient is asked to estimate the frequency of each item during the last week on a four-point scale ranging from 'not at all' to 'often'.

### *Anxiety and depression*

The HAD scale consists of two subscales, one assessing depression (seven items) and one anxiety (seven items). Subscale scores range from 0 (no distress) to 21 (maximum distress) (Zigmond and Snaith, 1983). Zigmond and Snaith (1983) suggested a score of 7 or less as indicative of a 'non-case', 8–10 as a 'doubtful case' and a score of 11 or more as a 'case'.

### Statistical analysis

Stepwise regression analyses were performed to determine how much of the variance in anxiety and depression at 6 months after the diagnosis was explained by anxiety, depression, subjective distress and coping close to the diagnosis. The extent to which it was possible to identify patients with moderate (doubtful cases) or high levels (cases) of delayed anxiety and depression was assessed in terms of sensitivity, specificity and overall predictive value.

Sensitivity expresses the correct proportion of identified cases (number of true positives/number of true positives plus number of false negatives) and specificity is the proportion of correctly identified non-cases (number of true negatives/number of true negatives plus number of false positives). One-factor ANOVA (repeated measures) were performed to determine changes over time and two-tailed (unpaired) *t*-tests were used for calculating differences in observed frequencies.

### RESULTS

### Prediction of anxiety and/or depression 6 months after diagnosis

Mean HAD scores at diagnosis were 4.0 for anxiety and 4.4 for depression. Patients who survived and were interviewed after 6 months had lower mean values of HAD depression (mean 3.7) in connection with the diagnosis than those who did not (mean 5.8, $t = 3.2$, $P < 0.01$). There was no corresponding difference in mean values of anxiety. There was a significant decrease over time in both mean HAD anxiety and depression (Table 2).

In a stepwise regression analysis, including the independent variables (data at diagnosis) HAD anxiety and depression, the MAC scale (fighting spirit, hopeless/helplessness, anxious preoccupation and fatalism) and the IES scale (intrusion and avoidance), HAD anxiety (31%) and depression (4%) accounted for a total of 35% of the variance in anxiety that was found 6 months later. For HAD depression 6 months after the diagnosis, 31% of the variance was explained by depression close to the diagnosis and 3% by hopeless/helplessness. When the 6-month scores for anxiety and depression were summarized, 37% of the variance was explained by depression and 4% by intrusion at diagnosis (Table 3). In an effort to avoid the multicollinearity problem, i.e. when variables are highly correlated, some of the coping scales were excluded from the analysis. This did not affect the pattern of results (data not shown). Inclusion of age, sex or state of illness (potentially cured/non-cured at diagnosis) in the multivariate analyses did not change the results, and no additional explanation was provided (data not shown).

### Prospective identification of anxiety and/or depression 'doubtful cases' or 'cases'

Of the patients who were alive 3 (non-cured patients) or 6 (potentially cured patients) months after the diagnosis, 24 (21%) scored as 'doubtful cases' or 'cases' for HAD anxiety and/or depression close to the diagnosis. Twelve of these patients still scored as 'doubtful cases' or 'cases' 3 or 6 months later. Close to the diagnosis, 89 (78%) patients scored as 'non-cases' and 87 (98%) of those did so 3 or 6 months later (Table 4). Using 'doubtful cases' or 'cases' for HAD anxiety or depression close to the diagnosis for the selection of patients who may need psychological intervention, i.e. score as 'doubtful cases' or 'cases' after 3 or 6 months, respectively, gives a sensitivity of 50% and a specificity of 98% (Model 1, Table 4).

An alternative model for the identification of patients at diagnosis for whom psychological intervention may be necessary is to employ 'doubtful cases' or 'cases' for HAD anxiety or depression together with scores above the cut-off level of 9 (medium to high subjective distress) for IES intrusion. This model (Model 2, Table 4) reaches a higher sensitivity (75%) while retaining a very high specificity (98%) compared with the use of the HAD scales alone (Table 4).

### DISCUSSION

Patient anxiety and depression close to the diagnosis were shown to explain approximately 35% of the variance in anxiety and

© Cancer Research Campaign 1999

depression that was found 6 months later. Thus, levels of anxiety and depression at diagnosis are predictive of a similar status 6 months later. The addition of data on coping strategies and subjective distress does very little to improve that prediction.

Multiple regression analyses for prediction yield information only about the proportion of variance explained by various independent variables. This kind of knowledge is not easily applied in everyday clinical decision-making about individual patients. For example, these analyses do not allow identification of individual patients at risk of delayed anxiety and depression. We therefore sought an alternative procedure with better applicability to clinical situations, namely, to identify prospectively patients scoring as 'doubtful cases' or 'cases'. Using 'doubtful cases' or 'cases' for HAD anxiety and/or depression to select patients at risk of prolonged psychological distress demonstrated that 50% of those selected may not be in great need of psychological intervention. Thus, half of patients will be offered an intervention unnecessarily because they spontaneously, or with the help of the regular care system, improve to such an extent that they no longer belong to the categories 'doubtful cases' or 'cases'. In the same way, inclusion of IES intrusion data will lead to unnecessary interventions for 25% of the patients. Practically no patients (2%) will be missed in either model.

Identification at diagnosis of patients at risk for elevated delayed levels of anxiety and depression should facilitate early psychological intervention for this group. The HAD scale has been shown to be a sensitive tool for the screening of psychiatric disorders among cancer patients (Razavi et al, 1990). Optimally, all newly diagnosed cancer patients who want professional psychological support should be offered this. However, an important consideration is then the cost of such support, in terms of both professional time and resources. Such considerations are particularly important as not all patients suffer from high levels of psychological distress close to the diagnosis. In addition, distress seems to decrease for most patients independently of psychological support from health care staff (Glanz and Lerman, 1992), as is also evident from the present data. If resources are limited, it may be economically impossible to intervene for all patients who express high levels of distress after diagnosis.

All adjuvant protocols, whether pharmacological or psychological, used to decrease the proportion from suffering a negative event imply overtreatment of a significant proportion. Ideally, a higher sensitivity than reached here would have been desirable. However, if 25% or even 50% of the patients were to receive 'unnecessary' psychological interventions, this would not result in high economic costs. The number of gastrointestinal cancer patients in need of psychological interventions (in this study defined as having elevated levels of HAD anxiety or depression) is rather limited. Also, several patients in adjuvant psychological protocols, mostly those not truly in need of the treatment, voluntarily drop out after one or two sessions (Hellbom et al, 1998).

Several factors have been suggested to influence the levels of psychological morbidity in cancer patients (Harrison and Maguire, 1994). Some of these are demographic factors (e.g. age, gender and marital status), pre-morbid psychological factors (e.g. self-esteem and past psychiatric history) and environmental factors (e.g. social support). These factors are consequently important for the physician to address, but as a complement, an easily completed questionnaire may be of advantage by helping the physician to detect patients at high risk for prolonged psychological distress.

The minimal additional value of the MAC scale as a predictor of later anxiety and depression may be explained by difficulties separating coping, measured by the MAC scale, from the outcomes of the coping efforts. In a preceding study (Nordin, 1997), we also found it difficult to separate coping as measured by the MAC scale, especially hopeless/helplessness and anxious preoccupation, from the outcome of the coping efforts, in this case HAD anxiety and depression. Thus, these scales may largely measure the same phenomena.

The use of the HAD scale, in combination with the IES intrusion subscale, to detect patients who are at risk for delayed anxiety and depression has not been tested before. Therefore, this study should be considered as exploratory, particularly as the number of patients scoring as 'doubtful cases' or 'cases' 3 or 6 months after diagnosis is small. If the results can be confirmed on larger patient series, this combination of scales could be practically useful.

In conclusion, levels of HAD anxiety and depression at diagnosis predict a similar status 6 months later. The addition of coping does very little to improve that prediction. The results also suggest that the HAD scale in combination with the IES intrusion subscale may be used as a tool for detecting patients at risk for prolonged psychological distress. Patients scoring as 'doubtful cases' or 'cases' for HAD anxiety or depression and above a cut-off of 9 in the intrusion subscale should be offered early psychological intervention. It should be noted that the scales are easy to handle for both the physician and the patients.

### ACKNOWLEDGEMENT

This research was made possible by a grant from the Swedish Cancer Society.

### REFERENCES

Andersen B (1992) Psychological interventions for cancer patients to enhance the quality of life. *J Consult Clin Psychol* **60**: 552–568

Burton M, Parker R, Farell A, Bailey D, Conneely J, Booth S and Elcombe S (1995) A randomized controlled trial of preoperative psychological preparation for mastectomy. *Psycho-Oncology* **4**: 1–19

Derogatis L, Marrow G, Denmann D, Piasetsky S, Schmale A, Heinrichs M and Carnicke C (1983) The prevalance of psychiatric disorders among cancer patients. *JAMA* **249**: 751–757

Fawzy F, Fawzy N, Hyun C, Elashoff K, Guthrie D, Fahey and Morton D (1993) Effects of an early structured psychiatric intervention, coping, and affective state on recurrence and survival 6 years later. *Arch Gen Psychiat* **50**: 681–689

Glanz K and Lerman C (1992) Psychosocial impact of breast cancer: a critical review. *Ann Behav Med* **14**: 204–212

Greer S, Moorey S, Baruch J, Watson M, Robertson B, Mason A, Rowden L, Law M and Bliss J (1992) Adjuvant psychological therapy for patients with cancer: a prospective randomised trial. *BMJ* **304**: 675–680.

Greer S, Moorey S and Watson M (1989) Patients adjustment to cancer (MAC) scale vs clinical ratings. *J Psychosom Res* **33**: 373–377

Harrison J and Maguire P (1994) Predictors of psychiatric morbidity in cancer patients. *Br J Psychiat* **165**: 593–598

Hellbom M, Brandberg Y and Sjödén P (1998) Individual psychological support for cancer patients: utilization and patient satisfaction. *Pat Ed Counsel* **34**: 247–256

Horowitz M (1993) Stress response syndromes and their treatment. In *Handbook of Stress. Theoretical and Clinical Aspects*, Goldberger L and Breznitz S (eds), pp 757–773. The Free Press: New York

Horowitz M, Wilner N and Alvarez W (1979) Impact of Event Scale: a measure of subjective stress. *Psychosom Med* **41**: 209–213

Leigh S, Wilson K, Burns R and Clark R (1995) Psychosocial morbidity in bone marrow transplant recipients: a prospective study. *Bone Marrow Trans* **16**: 635–640

Maguire G (1992) Improving recognition and treatment of affective disorders in cancer patients. In *Recent Advances in Clinical Psychology*, Granville-Grossman K (ed.). Churchill Livingstone, Edinburgh.

© Cancer Research Campaign 1999

Nordin K and Glimelius B (1997). Psychological reactions in newly diagnosed gastrointestinal cancer patients. *Acta Oncol* **36**: 803–810

Razavi D, Delvaux N, Farvacques C and Robaye E (1990) Screening for adjustment disorders and major depressive disorders in cancer in-patients. *Br J Psychiat* **156**: 79–83

Schwartz C, Daltroy L, Brandt U, Friedman R and Stolbach L (1992) A psychometric analysis of the Mental Adjustment to cancer scale. *Psychol Med* **22**: 203–210

Spijker AVT Trijsburg RW and Duivenvoorden HJ (1997) Psychological sequelae of cancer diagnosis: a meta-analytical review of 58 studies after 1980. *Psychosom Med* **59**: 280–293

Trijsburg R, Knippenberg van F and Rijpma S (1992) Effects of psychological treatment on cancer patients: a critical review. **54**: 489–517

Watson M (1983) Psychosocial intervention with cancer patients: a review. *Psychol Med* **13**: 839–846

Watson M, Fenlon D, McVey G and Fernandez-Marcos M (1996) A support group for breast cancer patients: development of a cognitive-behavioural approach. *Behav Cogn Psychoth* **24**: 73–81

Watson M, Greer S, Young J, Inayat Q, Burgess C and Robertson B (1988) Development of a questionnaire measure of adjustment to cancer: the MAC scale. *Psychol Med* **18**: 203–209

Zigmond A and Snaith R (1983) The Hospital Anxiety and Depression Scale. *Acta Psychiat Scand* **67**: 361–370

*© Cancer Research Campaign 1999*
